# Risk Factors for Complications after Reduction Mammoplasty: A Meta-Analysis

**DOI:** 10.1371/journal.pone.0167746

**Published:** 2016-12-09

**Authors:** Min-Xia Zhang, Chun-Ye Chen, Qing-Qing Fang, Ji-Hua Xu, Xiao-Feng Wang, Bang-Hui Shi, Li-Hong Wu, Wei-Qiang Tan

**Affiliations:** 1 Department of Plastic Surgery, The First Affiliated Hospital, College of Medicine, Zhejiang University, Hangzhou, Zhejiang Province, P.R China; 2 Department of Plastic Surgery, The Fourth Affiliated Hospital, College of Medicine, Zhejiang University, Yiwu, Zhejiang Province, P.R China; BG Trauma Center Ludwigshafen, GERMANY

## Abstract

**Background:**

Reduction mammoplasty (RM) is a proven method of treating macromastia, but the risk factors for postoperative complications have not been clearly identified. Through this meta-analysis, the authors aimed to identify the risk factors of RM complications.

**Methods:**

An extensive search of the literature describing complications after RM was performed using the PubMed Central, Embase, and Cochrane databases. The following risk factors were extracted: age, body mass index (BMI), tissue resection weight per breast (TRW), smoking and radiation therapy. Odds ratios (OR) were pooled with 95% confidence intervals (CI) to evaluate the relationship between these risk factors and complications after RM.

**Results:**

A total of 16 unique studies including 10 593 patients were included in the final analysis. It showed that there was a significant difference in complications in BMI ≥30 kg/m^2^ (OR 0.73; 95% CI: 0.61–0.89, p = 0.001) and smoking (OR 1.56; 95% CI: 0.98–2.49, p = 0.06). Infection in those with BMI ≥30 kg/m^2^ showed a significant difference (OR 0.68; 95% CI: 0.52–0.89, p = 0.004), as well as wound dehiscence in smokers (OR 2.73; 95% CI: 1.60–4.67, p = 0.0002) and infection in irradiated breasts (OR 20.38; 95% CI: 3.42–121.35, p = 0.0009). However, there was no significant difference in age ≥50 years (OR 0.96; 95% CI: 0.71–1.29, p = 0.78), combined TRW ≥1000 g (OR 1.04; 95% CI: 0.43–2.50, p = 0.93).

**Conclusions:**

BMI ≥30 kg/m^2^ and smoking increase the risk of complications. Persons who are obese or irradiated are more likely to develop infections, and smokers experienced a higher incidence of wound dehiscence than did nonsmokers. However, patients aged ≥50 years and TRW ≥1000 g are not associated with complications from RM.

## Introduction

Macromastia is a common problem among women, leading to disabling symptoms such as neck, back, and shoulder pain; inframammary maceration; heavy breathing during exercise; and great psychological burdens because of unaesthetic appearance. Reduction mammoplasty (RM) is an approved procedure for women with macromastia, which has effectively relieved existing symptoms with high satisfaction, even though complications have often occurred [1–4]. Risk factors for complications are major determinants in surgical planning. Effectively predicting and preventing complications in RM has become an important research field. However, no predictors have been clearly recognized.

Postoperative complications after breast reduction include infection, wound healing problems, scars, fat necrosis, seroma, lost nipples and reoperations. The complications occurred after breast reduction would be as high as 40 or 50% in reported studies [3, 5, 6]. Many studies have reported preoperative factors that impacts complication rates [7, 8]. Increased body mass index (BMI) is often deemed a critical risk factor for postoperative complications [5, 9, 10]. Some analyses provide contradictory suggestions [11–13], which may be due to small sample size. Other reported risk factors are age, smoking, tissue resection weight per breast (TRW), radiation therapy and so on [6, 14, 15].

Almost all the risk factors associated with complications were controversial and no published meta-analysis had investigated it. We therefore performed a meta-analysis of all published prospective and retrospective studies to evaluate the important risk factors in women with macromastia and gigantomastia and provide preliminary guidance for clinical treatment and prognosis.

## Materials and Methods

We prospectively defined the study objectives, search paramters, eligibility criteria, and analytical methods.

### Search strategy

The following electronic databases were searched: The PubMed Central, Embase, and Cochrane Library. They were searched for English language studies using the following headings and keywords: macromastia or gigantomastia. Also used were breast reduction, reduction mammaplasty or reduction mammoplasty. No limitation was put on the date of publication, which covered all previously published studies up to December 2015. In addition, selected study references and review articles were examined for further article sources.

### Eligibility criteria

The initial selection of studies was performed on the basis of titles and abstracts. Next, two investigators (Min-Xia Zhang and Chun-Ye Chen) independently screened the full text of each selected study using the following inclusion criteria: (1) the study must meet the definition of macromastia or gigantomastia; (2) RM as the only surgical procedure of interest; (3) measured complications of the incidence and risk factors of RM; (4) risk factors were BMI, age, TRW, smoking or radiation therapy, any one of which should be researched in the study; (5) Sufficient data on contrasting groups. Studies were excluded if they contained any one of the following exclusion criteria: (1) case reports, abstracts only, letters, comments or reviews; (2) studies with mixed gender or surgical procedures; (3) virginal, adolescent or pregnant macromastia. To avoid double publication, only the most informative or lastest study was included. This meta-analysis was performed in accordance with the Preferred Reporting Items for Systematic Reviews and Meta-Analyses (PRISMA) statement checklist.

### Data extraction

Data extraction was performed independently by two reviewers (Min-Xia Zhang and Chun-Ye Chen), and any disagreement concerning paper eligibility was resolved by discussion and consensus. The data covered the general characteristics of each study and the outcomes measured. In addition, studies were assigned a level of evidence score.

### Statistical analysis

Based on the amount of data available and on clinical relevance, five factors were analyzed including BMI, age, TRW, smoking and radiation therapy.

For each risk factor in our study, odds ratios (OR) and 95% confidence intervals (CI) were calculated for outcomes. A p value <0.05 was judged as statistically significant. Random-effects models were used depended on the heterogeneity of the studies included. Heterogeneity was analyzed with both the Chi squared test *I* square test, where p value <0.10 for the Chi squared and I^2^ ≥50% implied heterogeneity [16]. The forest plot was a graphic presentation of the analytic result.

Data was processed in Review Manager version 5.3 from the Cochrane Collaboration.

## Results

### Study identification and selection

**Fig 1:**
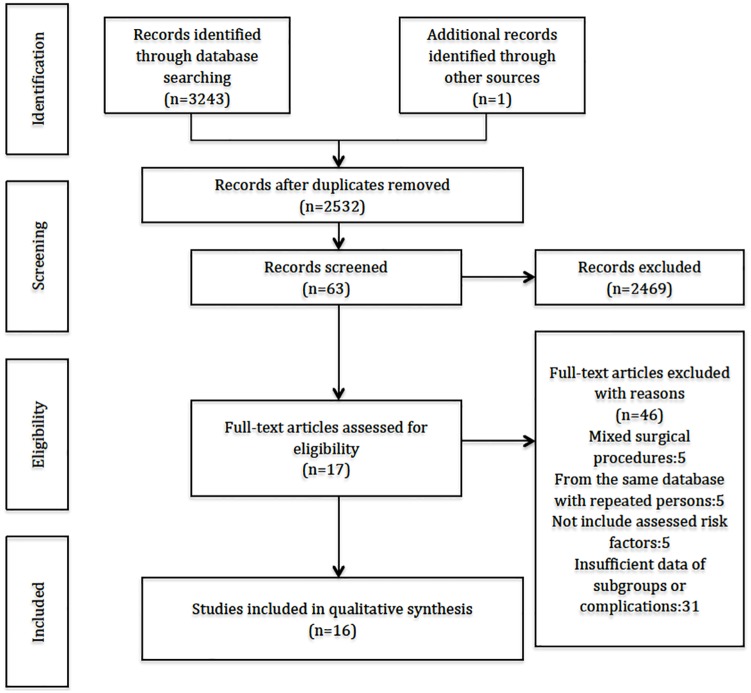
Preferred Reporting Items for Systematic Reviews and Meta-Analysis flow diagram, literature search, and selection process.

A total of 2532 records were identified by the initial database search, from which 63 full-text articles were retrieved for final review after screening titles and abstracts. Of these, 16 studies that met all predefined inclusion criteria were finally included in our meta-analysis [5, 6, 11, 14, 15, 17–27]. Fig 1 shows study selection through the processes of identification, screening, and eligibility (Fig 1). Evidence for and against each risk factor is stratified in Table 1.

**Table 1:** Literature summary of major risk factors for complications.

Risk Factor	Supporting Evidence	Refuting Evidence	Meta-Analysis OR (95% CI)	P value
BMI≥30 kg/m^2^	Shah R et al. 2011; Chun YS et al. 2012; Nelson JA et al. 2014 (obesity)	Cunningham BL et al. 2005; Schumacher HH et al. 2005; Kendall R et al. 2008; Roje Z et al. 2012; Guemes A et al. 2015; Setala L et al. 2009	0.73 (0.61–0.89)	0.001
Age≥50 yrs	None	Cunningham BL et al. 2005; Schumacher HH et al. 2005; Kendall R et al. 2008; Roje Z et al. 2012; Nelson JA et al. 2014 (age); Guemes A et al. 2015; Setala L et al. 2009	0.96 (0.71–1.29)	0.78
TRW≥1000 g	Cunningham BL et al. 2005; Shah R et al. 2011	Schumacher HH et al. 2005; Kendall R et al. 2008; Roje Z et al. 2012; Guemes A et al. 2015	1.04 (0.43–2.50)	0.93
Smoking	Schumacher HH et al. 2005; Chan LK et al. 2006; Bikhchandani J et al. 2007; Shah R et al. 2011; Deliaert AE et al. 2012; Roje Z et al. 2012	Cunningham BL et al. 2005; Kendall R et al. 2008; Guemes A et al. 2015	1.56 (0.98–2.49)	0.06
Radiation Therapy (for infection)	Dal Cin A et al. 2012 Parrett BM et al. 2010	Weichman KE et al. 2015	20.38 (3.42–121.35)	0.0009

BMI: body mass index; TRW: tissue resection weight per breast

### Study characteristics

Study characteristics for the 16 included studies are summarized in Table 2. These studies were published before December 2015. There were three prospective studies while the others were retrospective ones. Mean postoperative follow-up time was noted in nine studies [6, 11, 15, 21–26] and occurred from 1 to 32.9 months after surgery. Five studies [5, 17–19, 27] did not report follow-up time, and the last two studies [14, 20] only reported a range.

**Table 2:** Study Characteristics.

References	LOE	Design	Years	NO. Of patients (breasts)	Follow-up (mo)
Schumacher HH et al. 2005[20]	III	R-COH	2001–2002	71(118)	0.3–15.2
Cunningham BL et al. 2005[6]	II	P-COH	NR	179(NR)	8
Chan LK et al. 2006[5]	III	R-COH	2002–2004	169(338)	NR
Bikhchandani J et al. 2007[19]	III	R-COH	1999–2004	402(762)	NR
Kendall R et al. 2008[14]	III	R-COH	1996–2006	179(358)	1–12
Setala L et al. 2009[11]	III	R-COH	1998–2003	273(546)	6
Shah R et al. 2011[17]	III	R-COH	1999–2004	306(NR)	NR
Chun YS et al. 2012[18]	III	R-COH	1995–2007	675(1350)	NR
Roje Z et al. 2012[22]	III	R-COH	1995–2011	59(117)	6
Deliaert AE et al. 2012[24]	II	P-COH	2006–2007	43(NR)	1
Nelson JA et al. 2014[21]	III	R-COH	2005–2011	4545(NR)	1
Nelson JA et al. 2014[15]	III	R-COH	2005–2010	3537(NR)	1
Guemes A et al. 2015[23]	II	P-COH	2012–2013	121(NR)	1
Dal Cin A et al. 2012[25]	III	R-COH	1980–2007	9(18)	32.9
Parrett BM et al. 2010[26]	III	R-COH	2004–2008	12(24)	10
Weichman KE et al. 2015[27]	III	R-COH	2001–2003	13(26)	NR
Total				10593	

R-COH: retrospective cohort; P-COH: prospective cohort; LOE: level of evidence; NR: not reported

### Patient and treatment characteristics

The study set included altogether 10 593 patients whose demographic features (age, BMI, smokers, TRW, operating time and techniques) are displayed in Table 3, with some studies providing detailed information and others not. The overall incidence of complications of 11 studies [6, 11, 14, 15, 17, 18, 20–24] was 11.0% (ranged 5% to 56%), while the rest [5, 19, 25–27] reported only some specific complications, lacking the total quantities. Various operative techniques were used, in which the most frequent technique was the inferior pedicle with an average percentage of 46.0%. The medial pedicle and central mound technique were exclusively used in 2 studies, respectively. The vertical scar pattern was also used frequently, as well as the superomedial pedicle and free nipple graft.

**Table 3:** Patient and Treatment Characteristics.

References	Ag (yr)	BMI (kg/m^2^)	Smokers(%)	Tissue resection weight per breast(g)	operating time (min)	Techniques (%)
Schumacher HH et al. 2005[20]	39.0	27.5	33.8	648.0	NR	NR
Cunningham BL et al. 2005[6]	39.4	29.7	11.2	814.0	121–154.4	NR
Chan LK et al. 2006[5]	36.2	26.7	38.5	713.8	NR	IP:63.3; VS:4.1;SP:29.0; FNG:3.6
Bikhchandani J et al. 2007[19]	34.3	28.9	27.9	NR	NR	IP:most
Kendall R et al. 2008[14]	35.0	34.0	4.2	NR	NR	IP:74.9; VS:4.5; FNG:20.6
Setala L et al. 2009[11]	43.0	28.0	NR	730.5	NR	NR
Shah R et al. 2011[17]	36.8	27.6	NR	NR	NR	FNG:19.6; Others:80.4
Chun YS et al. 2012[18]	37.5	31.0	1.3	848.0	131.0	IP:80.7; VS:0.6;Others:18.7
Roje Z et al. 2012[22]	47.0	28.0	22.0	1057.0	111	IP:10.2; VS:72.9; SP:10.2; FNG:6.7
Deliaert AE et al. 2012[24]	35.9	26.2	30.2	437.3	NR	MP:100
Nelson JA et al. 2014[21]	NR	NR	11.7	NR	173.1	NR
Nelson JA et al. 2014[15]	43.2	31.6	12.1	NR	180.7	NR
Guemes A et al. 2015[23]	40.7	29.6	34.7	NR	NR	IP:100
Dal Cin A et al. 2012[25]	56.2	30.0	NR	577.3	NR	IP:88.9; SP11.1
Parrett et al. 2010[26]	57.0	29.9	0	452.5	NR	IP:41.7; Others:58.3
Weichman KE et al. 2015[27]	50.2	26.8	0	320.5	NR	CMT:100
Average	43.7	30.3	13.3	690.3	172.5	IP:46.0; VS:8.2; SP:5.0; FNG:5.1; MP:10.0;CMT:10.0; Others:15.7

BMI: body mass index; NR: not reported; IP: inferior pedicle; VS: vertical scar; SP: superomedial pedicle; FNG: free nipple graft; MP: medial pedicle; CMT: central mound technique

### Assessment of risk factors of complications

The risk factors with sufficient data of complications available for meta-analysis were BMI, age, TRW, smoking and radiation therapy. The results were expressed as ORs, 95% CIs and p-values.

#### BMI ≥30 kg/m^2^

**Fig 2:**
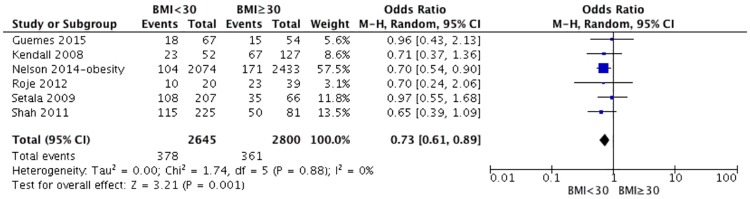
Correlations between BMI and complications.

**Fig 3:**
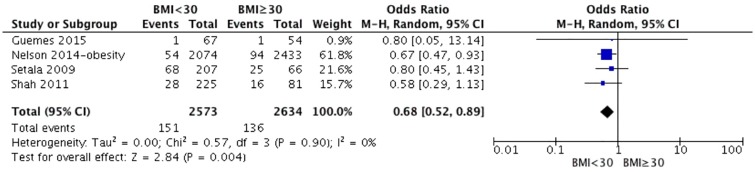
Correlations between BMI and infections.

We first divided the patients into two groups: non-obese (BMI <30 kg/m^2^) and obese (BMI ≥30 kg/m^2^). BMI ≥30 kg/m^2^ was the strongest risk factor for overall complications in our meta-analysis based on 6 related studies [11, 14, 17, 21–23]. The test for heterogeneity was not significant (p for heterogeneity = 0.88; I^2^ = 0%). The aggregated results suggest that BMI ≥30 kg/m^2^ was highly associated with a significant increase in the overall incidence of complications after RM (OR 0.73; 95% CI: 0.61–0.89, p = 0.001) (Fig 2). Moreover, the aggregated results of the 4 studies [11, 17, 21, 23] suggest that BMI ≥30 kg/m^2^ was highly associated with a significant increase in the incidence of infection (OR 0.68; 95% CI: 0.52–0.89, p = 0.004) (Fig 3). The test for heterogeneity was not significant (p for heterogeneity = 0.9; I^2^ = 0%).

#### Age ≥50 years

**Fig 4:**
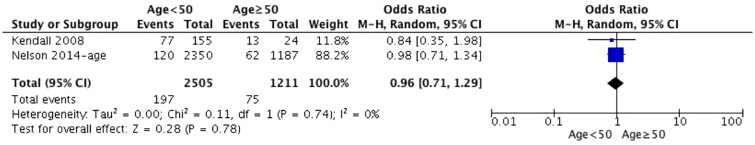
Correlations between age and complications.

Two articles [14, 15] reported the relationship between age at surgery (<50 or ≥50) and complications after RM. The test for heterogeneity was not significant (p for heterogeneity = 0.74; I^2^ = 0%). The aggregated results of the 2 studies suggest that age ≥50 years was not associated with a significant increase in the overall incidence of complications after RM (OR 0.96; 95% CI: 0.71–1.29, p = 0.78) (Fig 4).

#### TRW ≥1000 g

**Fig 5:**
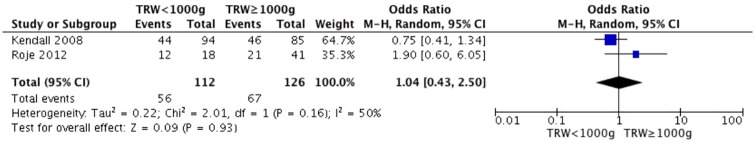
Correlations between tissue resection weight per breast and complications.

Two articles [14, 22] reported the relationship between TRW (<1000 g or ≥1000 g) and complications after RM. The test for heterogeneity was significant (p for heterogeneity = 0.16; I^2^ = 50%). Using the random-effect model, the aggregated results suggest that TRW ≥1000 g was not associated with a significant increase in the overall incidence of complications after RM (OR 1.04; 95% CI: 0.43–2.50, p = 0.93) (Fig 5).

#### Smoking status

**Fig 6:**
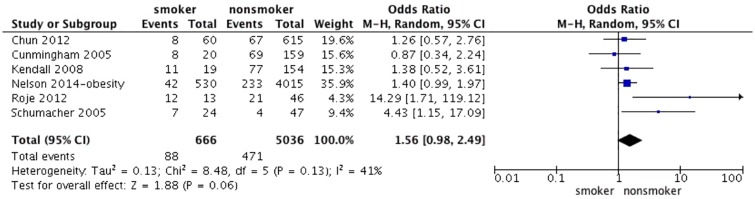
Correlations between smoking and complications.

**Fig 7:**
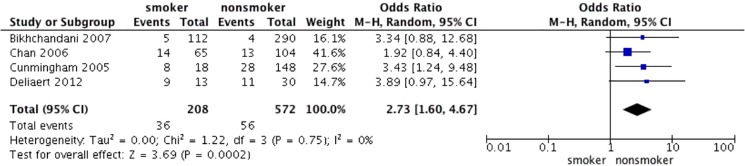
Correlations between smoking and wound dehiscence.

We combined 6 studies [6, 14, 18, 20–22] concerning smoking and complications after breast reduction. The test for heterogeneity was significant (p for heterogeneity = 0.13; I^2^ = 41%). Using the random-effect model, the aggregated results suggest that smoking was highly associated with a significant increase in the overall incidence of complications (OR 1.56; 95% CI: 0.98–2.49, p = 0.06) (Fig 6). In addition, smoking was also highly associated with a significant increase in the incidence of wound dehiscence after RM of 4 studies [5, 6, 19, 24] (OR 2.73; 95% CI: 1.60–4.67, p = 0.0002) (Fig 7). The test for heterogeneity was not significant (p for heterogeneity = 0.75; I^2^ = 0%).

#### Radiation therapy

**Fig 8:**
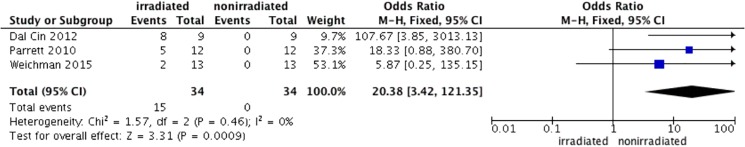
Correlations between radiation and infection.

**Fig 9:**
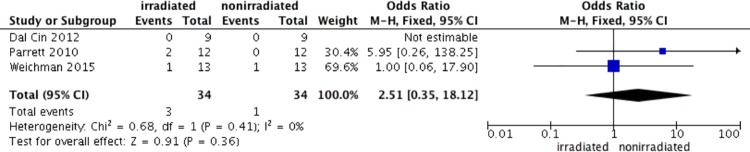
Correlations between radiation and fat necrosis.

We finally selected 3 studies [25–27] reporting radiation therapy and complications after RM. The test for heterogeneity was not significant (p for heterogeneity = 0.46; I^2^ = 0%). The aggregated results suggest that breast reduction after radiation lead to a significant increase in the incidence of infection (OR 20.38; 95% CI: 3.42–121.35, p = 0.0009) (Fig 8). However, the fat necrosis after RM was not significant between irradiated and nonirradiated breasts (OR 2.51; 95% CI: 0.35–18.12, p = 0.36) (Fig 9). The test for heterogeneity was not significant (p for heterogeneity = 0.41; I^2^ = 0%).

### Publication bias

For observational studies, we applied the Risk of Bias Assessment tool for Nonrandomized Studies (RoBANS), which was compatible with the Cochrane risk-of-bias tool.

## Discussion

Even though women undergoing breast reduction were generally young and healthy, postoperative complications were relatively common, with an incidence of 14%-53% in reported studies [6, 17, 28], of which the risk factors associated with complications after RM were controversial, so we performed a meta-analysis to evaluate the important risk factors.

Increased BMI was the strongest predictor of complications among patients with breast reduction in our meta-analysis. The patients’ weights were categorized as follows: a BMI equal to or less than 18.5 kg/m^2^ was classified as “underweight”; a BMI between 18.5 and 24.99 kg/m^2^ was “normal weight”; a BMI between 25 and 29.99 kg/m^2^ was “overweight” and a BMI of 30 kg/m^2^ or more was “obese” (in line with the WHO general classification of obesity) [29]. Based on their BMIs, the patients were divided into two groups (underweight, normal, and overweight formed the “non-obese” group; the remainder comprised the “obese” group) [23]. The vast majority of studies concluded that breast reduction surgery in the obese population had a significantly higher rate of complications than in the nonobese population [10, 17], although there was some evidence to the contrary [11, 23, 30]. One significant limitation with the majority of these studies, however, was that they were mostly single institution case series with homogeneous patient populations [21]. Our assumption that surgical technique might be the biggest element resulted in contrary conclusions in different studies. What is more, the results of our meta-analysis indicated that obese patients were more likely to experience complications, especially infection, than the nonobese, which was based on a large number of patients. On a biological level, the relative vascularity of adipose tissue makes this tissue more vulnerable to infection. Obese women have been shown to have impaired capillary recruitment and acetylcholine-mediated vasodilation [31]. In addition, obese individuals have significantly increased transepidermal water loss and erythema compared to controls [21]. Our results suggest that full disclosure of high postoperative complication rates in patients with a higher BMI is essential and that they should also be well informed that the chances of infection are also higher than in nonobese patients. If avoidable, breast reduction surgery should not be performed on obese patients unless they lose weight.

Our mate-analysis demonstrated that age at surgery ≥50 years bears no relationship to complications in RM compared with younger patients <50 years. According to a positive study by Shermak MA et al.[32], we divided the patients into two groups with a boundary of 50 years. In fact, Shermak MA et al.[32] put forward that age older than 50 years impaired breast reduction outcomes, particularly infection, and might negatively impact wound healing. However, Nelson JA et al.[15] suggested that RM could be performed safely on older patients with proper patient selection. This assertion is supported by other studies [11, 14] whose results were similar to ours. The results of this meta-analysis suggest that RM can be safely performed on elderly patients. However, patients with severe comorbidities or high BMI should not be admitted, whether young or old. Therefore, appropriate patient selection and counseling are essential before surgery.

TRW ≥1000 g was not a significant factor in the overall incidence of complications compared with smaller resections. We had arbitrarily set the crossover point between small and large reductions at 1000 g of tissue per breast [33]. Zubowski R et al.[34] determined that the likelihood of developing complications increased linearly as the reduction size increased, and Dabbah A et al.[3] noted that there was a considerable increase in the number of complications when reductions over 1000 g per breast were performed. Similar results were found by Shah R et al.[17] and Cunningham BL et al.[6]. In contrast, Roje Z et al.[22] concluded that there was no significant relationship between patient complications and resection weight of breast parenchyma. Our meta-analysis with a large sample size implied that surgeons might properly finish the RM procedure to relieve a patients’ extreme symptoms, and omitted mentioning how much tissue was resected. However, more resection than average was not admitted in order to maintain aesthetics.

Another strong risk factor for complications after RM in our meta-analysis was smoking. Many other studies supported our results of the effect of smoking status on complication rates, such as Schumacher HH et al.[20], Roje Z et al.[22] and others. However, Kendall R et al.[14] found no statistically significant increase in complications in smokers, as did Cunningham BL et al.[6] and Guemes A et al.[23]. We suspected that their definition of smokers was different, which might have brought about different results. Furthermore, our results indicated that smokers had higher rates of experiencing wound dehiscence than did nonsmokers. Wound-healing problems were the most frequent complications among smokers according to previous studies [5, 19, 24]. Smoking contributed a thrombogenic state, induced endothelial wall damage, inhibited capillary blood flow, and released catecholamines, which were responsible for complications [35]. In conclusion, our results implied that it was important to stop smoking before breast reduction surgery to reduce complication rates. We suggest that it is better to quit smoking for 6 months or more before surgery and a shorter time might be ineffective.

Although smoking was significant for complications in our meta-analysis (p = 0.06), heterogeneity was also significant (I^2^ = 41%), so we used the random-effect model and sensitivity analysis to evaluate the source of high heterogeneity. The results of sensitivity analysis showed that smoking was also highly associated with complications (p = 0.02) with a low heterogeneity (I = 0%) when excluding one study of Roje Z et al.[22]. We systematically and comprehensively assessed the study characteristics, patient and treatment characteristics and any differences from five other studies [6, 14, 18, 20, 21], but found no source of heterogeneity for the excluded study. It was reasonable for us to believe that the result was credible because it was strongly supported by available evidence.

Radiation therapy increased the postoperative infection rates, but it was not associated with fat necrosis in our meta-analysis. Several investigators had reported significantly increased rates of complications in patients treated with radiation, and some, including Dal Cin A et al.[25] and Parrett BM et al.[26], considered radiation therapy a contraindication to RM. We presumed that it was due to the known harmful effects of radiation. However, some recent researches implied that the postoperative complications were similar in both irradiated and nonirradiated breasts. For example, Weichman KE et al.[27] suggested that RM could be performed safely after irradiation. We hypothesized it may be attributed to their surgical procedure of central mound technique. In conclusion, what our meta-analysis suggests is that RM should not be performed on irradiated patients unless they are carefully selected. The central mound technique might be a good choice for surgeons.

In this meta-analysis, we rudimentarily understood which population was more likely to have complications after breast reduction, how we developed a better surgical strategy before operating, what we focused on intraoperation and what measures should be taken to minimize the occurrence of postoperative complications. This was preliminary a guidance for selecting patients of RM treatment. As a minimum, it helped pave the way, for future studies of complications.

We recognized several limitations in our study. First, some factors, such as preoperative breast volume, nipple elevation, surgical techniques and diabetes mellitus, which might be post-RM risk factors for complications were not analyzed because related studies were few and we could not reach a pooled result. Second, many possible risk factors (such as techniques) are not mutually exclusive, which might have led to overestimating a complication risk. This study was not designed to provide independent risk factors for complications, for the reporting data was insufficient to do a multivariate analysis. Third, this meta-analysis was an observational study so that a randomized controlled trial was necessary. However, performance of any such study would be difficult. Consequently, there was marked heterogeneity among risk factors. So we attempted to account for heterogeneity using a random-effect model for meta-analysis when the Cochran Q statistic was significant, and find the source of high heterogeneity using sensitivity analysis. Finally, perhaps the biggest limitation of this meta-analysis and an important target for future research was the inability to perform outcome subgroup analyses by operative technique.

## Conclusions

This meta-analysis demonstrated that BMI ≥30 kg/m^2^ and smoking status are risk factors for complications after RM. Moreover, BMI ≥30 kg/m^2^ and radiation therapy showed a statistically higher incidence of infection and smokers acquired higher significant rates of wound dehiscence. However, age ≥50 years and TRW ≥1000 g are not associated with post-RM complications. Further large-scale, well-designed studies are urgently needed.

## Supporting Information

S1 File PRISMA 2009 flow diagram. (DOC)

S2 File PRISMA 2009 checklist. (DOC)

## Abbreviations

BMI: body mass index
RM: reduction mammoplasty
TRW: tissue resection weight per breast
