# Supplementary material for: Risk Factors for Complications after Reduction Mammoplasty: A Meta-Analysis
Source: PLoS One. 2016 Dec 9;11(12):e0167746. doi: 10.1371/journal.pone.0167746 (PMC5147968; doi:10.1371/journal.pone.0167746)
Supplement: S1 File — (DOC) [file pone.0167746.s001.doc]

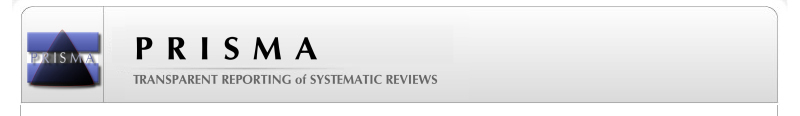
**PRISMA 2009 Flow Diagram**

**Screening**

**Included**

**Eligibility**

**Identification**

Records identified through database searching
(n = 3243)

Additional records identified through other sources
(n = 1)

Records after duplicates removed
(n = 2532)

Records screened
(n = 63)

Records excluded
(n = 2469)

Full-text articles assessed for eligibility
(n = 17)

Full-text articles excluded, with reasons
(n = 46)

Studies included in qualitative synthesis
(n = 16)

Studies included in quantitative synthesis (meta-analysis)
(n = 16)
